# Genome-wide association analysis of GAW17 data using an empirical Bayes variable selection

**DOI:** 10.1186/1753-6561-5-S9-S5

**Published:** 2011-11-29

**Authors:** Vitara Pungpapong, Libo Wang, Yanzhu Lin, Dabao Zhang, Min Zhang

**Affiliations:** 1 Department of Statistics, Purdue University, West Lafayette, IN 47907, USA

## Abstract

Next-generation sequencing technologies enable us to explore rare functional variants. However, most current statistical techniques are too underpowered to capture signals of rare variants in genome-wide association studies. We propose a supervised coalescing of single-nucleotide polymorphisms to obtain gene-based markers that can stably reveal possible genetic effects related to rare alleles. We use a newly developed empirical Bayes variable selection algorithm to identify associations between studied traits and genetic markers. Using our novel method, we analyzed the three continuous phenotypes in the GAW17 data set across 200 replicates, with intriguing results.

## Background

With the advent of next-generation sequencing, rare variants such as single-nucleotide polymorphisms (SNPs) with a minor allele frequency (MAF) less than 5% are getting more attention in genome-wide association studies (GWAS). Because of the small variance at a locus with a single rare allele, it is difficult to detect the allele’s association with the phenotype of interest. One approach to tackling this problem is to collapse multiple rare SNPs within a defined region and treat them as a single predictor in the model. Known genetic regions are used in the collapsing process to get gene-based markers. Penalized orthogonal-components regression (POCRE) [1] is used to perform this task.

Genome-wide association studies are challenged by the “curse of dimensionality”; that is, a large number of SNPs are genotyped (large *p*) from a small number of biological samples (small *n*). As a result, an increasing effort has been devoted to selecting variables in high-dimensional data. One strategy for dealing with variable selection is through the thresholding concept. Empirical Bayes thresholding [2,3] was proposed to estimate sparse sequences observed in Gaussian white noise. Here, we use the empirical Bayes thresholding method to select variables in linear regressions with efficient implementation. Final models are obtained by entering gene-based markers and environmental factors possibly associated with the phenotype of interest. All analyses are based on three continuous phenotypes in the GAW17 data set across 200 replicates.

## Methods

### Data set

The genome-wide association of the three continuous phenotypes (Q1, Q2, and Q4) in the GAW17 data set [4] was investigated. All analyses presented here are based on the genotype of 697 unrelated individuals. The genotype data were recoded into counts of minor alleles using PLINK [5]. The other three traits (Age, Sex, and Smoke) were used in the model to consider the environmental effects. The analyses were performed for all 200 replicates.

### Supervised coalescing of SNPs in a genetic region

The GAW17 data consist of 3,205 autosomal genes with 24,487 SNPs, where only 3,132 SNPs (12.79%) have MAF ≥ 0.05. A large proportion of these rare variants present challenges for statistical analyses to detect their associations to a phenotype of interest when these rare variants are considered individually. Thus we use a gene-based coalescing method to collapse SNPs that reside within the same gene. Considering a causal gene, it is natural to assume that not all SNPs in the genetic region are necessary to be causal. Hence we used POCRE in the coalescing process. Because POCRE can achieve both variable selection and dimension reduction simultaneously, it has advantages in grouping highly correlated predictors and in giving adaptive sparse linear combinations of the original predictors. For the *k*th genetic region, consider a regression model:(1)

where *l_k_* is the number of SNPs residing in the *k*th gene, **Y** is an *n*-vector of phenotype, and  is a design matrix consisting of SNPs residing in the *k*th gene. Assume that both **Y** and **X***_k_* are centralized (*τ_k_* = 0 in Eq. (1)). Starting with , POCRE sequentially constructs components  such that  is orthogonal to . The loading *ω_m_*, *m* ≥ 1, is obtained as *γ*/||*γ*||, with *γ* minimizing(2)

where *g_λ_*(*γ*) is a penalty function with a tuning parameter *λ*. Zhang and colleagues [1] used the empirical Bayes thresholding method proposed by Johnstone and Silverman [2,3] to introduce a proper penalty function, which provides adaptive sparse loadings of orthogonal components.

POCRE is a supervised learning method that needs the information of both genotype and phenotype to build a model. In the GAW17 data set, the genotype is held fixed but the phenotype varies across 200 replicates. To overcome potential overfitting in the model-building process, we selected one replicate as a training set to obtain the sparse coefficients of SNPs in each genetic region, and we then applied the results from POCRE to data in another replicate. In practice, when only one data set is available, cross-validation can be performed to select a tuning parameter to alleviate overfitting.

### Empirical Bayes variable selection

In the variable selection process, 3,205 gene-level markers acquired from the coalescing process and the other three traits (Age, Sex, and Smoke) were put into the model. The reason for putting Age, Sex, and Smoke in the model is the lack of knowledge about whether these three traits are associated with the studied trait. If some variables are known to be associated with the studied trait, then a regression model can be fitted with these known factors, with the residuals taken as new responses in the variable selection process. Empirical Bayes variable selection (EBVS) was proposed to obtain a final model. The EBVS algorithm works well in fitting a large-*p*, small-*n* regression model:(3)

where *p* is the number of predictors, **Y** is an *n*-vector of phenotype, and  is a design matrix. By further assuming that **Y** is centralized and **X** is standardized (*μ* = 0 in Eq. (3)), the EBVS puts the following mixture prior distribution to model the sparsity of *β_j_*:(4)

where  if  and  otherwise; and , following Johnston and Silverman [2,3]. Data-driven optimal values for *ω* and *a* were obtained to achieve adaptivity to sparseness and shape of prior distribution of *β_j_*, respectively. With current values of **β** and σ, the optimal values for *ω* and *a* are obtained as the values that maximize their full conditional distribution functions, *P*(*ω*|**β**,σ) and *P*(*a*|**β**,σ), respectively. **β** as the posterior median is then updated. The iterative procedure for updating **β** and hyperparameters is carried out until convergence. With this mixture prior distribution, EBVS gives a sparse solution for **β**.

## Results

The results of analyzing Q1 are shown in Table 1, which lists both genetic and environmental components identified to have nonzero effects in at least 5 out of 200 replicates. Among 200 replicates, four genes were identified as having nonzero effects: *FLT1* in 200 replicates, *KDR* in 53 replicates, *ARNT* in 12 replicates, and *RIPK3* in 6 replicates. The first three genes are true causal genes, but *RIPK3* is not. Another two environmental factors, Age and Smoke, are included in the final model across all 200 replicates. Because all the phenotypes in the GAW17 data set are simulated to be influenced by SNP-based markers, the gene-based results are transformed into SNP-based results, and we find that 23 out of 39 causal SNPs detected have nonzero effects. Eleven of these SNPs affiliate with *FLT1*. Ten of them are within the *KDR* region, and two of them are in the *ARNT* region.

**Table 1:** Identified genes and covariates in at least 5 out of 200 replicates for Q1

Gene/covariate	Average of ^a^	SD^a^	Frequency
Age	0.01667	0.00154	200
Smoke^b^	0.49877	0.06437	200
*FLT1*	0.78316	0.10969	200
*KDR*	0.65308	0.16401	53
*ARNT*	0.79018	0.30581	12
*RIPK3*	0.87993	0.28302	6

^a^ The average of  and its standard deviation are calculated on the basis of replicates whose component has a nonzero coefficient.

^b^ Smoke is coded as 1 for smokers and 0 for nonsmokers.

In addition, another 98 noncausal genes were identified. All of these genes were identified in only one or two out of 200 replicates, which might be due to noise. Another causal gene, *VEGFC*, was also found and included in the final model in two replicates. However, after transforming gene-based results into SNP-based results, none of the true causal SNPs affiliating to *VEGFC* were identified.

**Figure 1:**
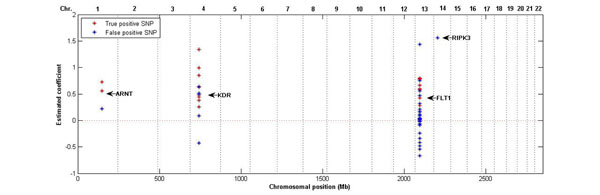
**Identified SNPs in at least 5 out of 200 replicates for Q1.** The *x*-axis indicates the chromosomal position of each SNP. The *y*-axis represents the frequency at which SNPs were identified as having nonzero effects across 200 replicates. Red dots represent true positives, and blue dots represent false positives.

**Figure 2:**
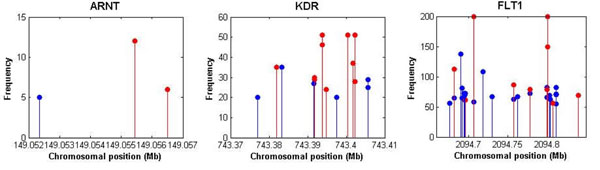
**Identified SNPs for Q1 within *ARNT*, *KDR*, and *FLT1* genetic regions. **The frequencies of identified SNPs within three genetic regions, *ARNT*, *KDR*, and *FLT1*, are shown. The *x*-axes indicate the chromosomal position of each SNP. The *y*-axes represent the frequency at which SNPs were identified as having nonzero effects across 200 replicates. Red dots represent true positives, and blue dots represent false positives.

For the SNPs identified in at least 5 out of 200 replicates, we plotted the frequencies of identified SNPs across 200 replicates against chromosomal position (Figure 1). In Figure 1, many of the identified SNPs are false positives, even though they affiliate to the causal genes. Figure 2 provides the plots of frequencies within three causal genetic regions: *FLT1*, *KDR*, and *ARNT*. Considering only genetic components, false-positive and false-negative rates were calculated for both gene-based and SNP-based results. Using the gene-based results obtained from EBVS, we calculated the average false-positive and false-negative rates across 200 replicates as 0.3067 (± 0.2634) and 0.0024 (± 0.0002), respectively. For the SNP-based results across 200 replicates, the average false-positive rate was 0.5819 (± 0.1708) and the average false-negative rate was 0.0012 (± 0.0002). The number of false-positive selections is not negligible and it is higher in SNP-based results. This is because identified gene-based markers include noncausal SNPs during the coalescing process to obtain gene-level markers.

Table 2 lists genes associated with Q2 that were found to have nonzero effect in at least 5 of the 200 replicates. Note that there are only two genes in this list and that their corresponding frequencies are low among the 200 replicates: *VNN1* (12 replicates) and *VNN3* (7 replicates). The low frequencies result from the low residual heritability of Q2 (0.29), which makes it difficult to detect any genetic signal. Moreover, Q2 was found to not be influenced by any environmental factors.

**Table 2:** Identified genes and covariates in at least 5 out of 200 replicates for Q2

Gene/covariate	Average of ^a^	SD^a^	Frequency
*VNN1*	1.35707	0.31121	12
*VNN3*	0.99105	0.17755	7

^a^ The average of  and its standard deviation are calculated on the basis of replicates whose component has a nonzero coefficient.

**Figure 3:**
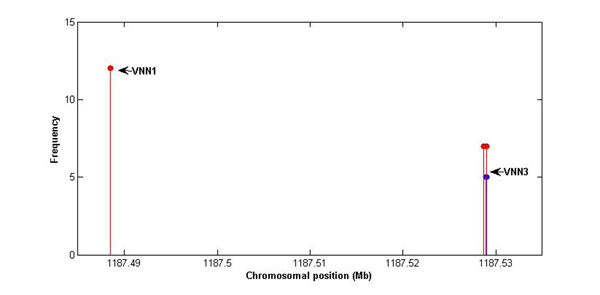
**Identified SNPs in at least 5 out of 200 replicates for Q2.** The *x*-axis indicates the chromosomal position of each SNP. The *y*-axis represents the frequency at which SNPs were identified as having nonzero effects across 200 replicates. Red dots represent true positives, and blue dots represent false positives.

For the true discoveries of SNP-level markers, 32 out of 72 true causal SNPs have been detected to have nonzero effects. However, the frequency of many true causal SNPs is 1. Only five of identified SNPs have frequencies greater than 5 (Figure 3): four of them affiliate to *VNN3* and one affiliates to *VNN1*. Both *VNN1* and *VNN3* are within the 6q23.2 region displayed in Figure 3. The average false-positive and false-negative rates for gene-based results across 200 replicates are 0.0625 (± 0.2238) and 0.004 (± 0.0001), respectively. For SNP-based results, the average false-positive rate is 0.0727 (± 0.2309) and the false-negative rate is 0.0029 (± 0.0001). The difficulty of detecting effects in a trait with a low residual heritability results in a low false-positive rate, and many false negatives were found here.

For Q4, all environmental factors, Age, Sex, and Smoke, have influences on this trait. Among 200 replicates, Age and Smoke were included in the final model in all 200 replicates, whereas Sex was included in the final model in 199 replicates (Table 3). Our results show that Q4 decreases with age, is higher in males, and is lower in smokers. In the GAW17 simulation, there is no genetic component influencing Q4. However, the analyses found 15 genes identified to have a nonzero effect, but all of them were detected in only one among 200 replicates. The average false-positive rates among 200 replicates are 0.0350 (± 0.1842) and 0.0300 (± 0.1710) based on gene-based and SNP-based results, respectively.

**Table 3:** Identified genes and covariates in at least 5 out of 200 replicates for Q4

Gene/covariate	Average of ^a^	SD^a^	Frequency
Age	–0.04591	0.00064	200
Smoke^b^	–0.36779	0.04127	200
Sex^c^	0.22870	0.03260	199

^a^The average of  and its standard deviation are calculated on the basis of replicates whose component has a nonzero coefficient.

^b^Smoke is coded as 1 for smokers and 0 for nonsmokers.

^c^Sex is coded as 1 for males and 0 for females.

## Discussion

With the next-generation sequencing technology, many rare variants or low-frequency SNPs can be detected. The customary criteria for MAF in data preprocessing (i.e., MAF ≥ 0.05) in GWAS is not appropriate in this situation. One possible solution is to reduce the cutoff point of MAF. Although this approach can be done easily, it is difficult to determine the optimal cutoff point. With too big a cutoff point, the majority of rare variants are discarded in analyses and little is gained from the next-generation sequencing data. With too small a cutoff point, most SNPs are included in a model, presenting challenges in statistical analyses for detecting signals of rare variants.

We grouped both common and rare variants in the same genetic region into a gene-based marker using POCRE. POCRE has a variable selection property that assumes that not all SNPs in a genetic region contribute to a gene-based marker. Although this assumption is realistic, the variable selection property of POCRE might rule out true causal SNPs in the coalescing process. On the other hand, the coalescing process might include noncausal SNPs, resulting in a false positive when the gene is identified to have nonzero effect by EBVS. Better techniques to combine SNPs into gene-based markers need to be further studied to overcome the challenges in the next-generation sequencing.

Another challenge in analyzing the GAW17 data is signal detection for a trait with low heritability. It is well known that it is difficult to identify nonzero effects in GWAS for a trait with low heritability. However, true causal rare variants worsen the situation and make the variants more difficult to detect. Better strategies need to be further explored in GWAS to tackle the problem of a low heritability trait with rare variants.

## Conclusions

In this study, we proposed using POCRE to coalesce common and rare variants in the same gene into a gene-level marker and applied the newly developed empirical Bayes variable selection to detect the association between markers and three continuous phenotypes in the GAW17 data set: Q1, Q2, and Q4. With a large number of predictors, the newly developed empirical Bayes approach not only selects important variables into the model but also estimates the effect sizes of nonzero predictors simultaneously.

Our results show that combining both common and rare variants into gene-level markers can increase the power to detect their signals. In fact, many identified true causal SNPs have MAF = 0.000717 or have variants that are found in only one individual. Nevertheless, there are still a number of false negatives. Based on GAW17 data, we notice that false negatives occur when only a few causal SNPs are present in the genetic region. When the size of causal SNPs in the gene region is moderate, it is still challenging to detect true signals when most of the causal SNPs are rare variants. As shown in our analysis, causal SNPs with higher MAFs can be identified more frequently than causal SNPs with lower MAFs.

## Competing interests

The authors declare that there are no competing interests.

## Authors’ contributions

VP and MZ designed the study, and VP performed the statistical analysis as well as drafted the manuscript. LW and YL carried out the preprocessing of the data. DZ and MZ conceived the study, reviewed, and edited the manuscript. All authors read and approved the final manuscript.
